# Supervised machine learning algorithms to predict the duration and risk of long-term hospitalization in HIV-infected individuals: a retrospective study

**DOI:** 10.3389/fpubh.2023.1282324

**Published:** 2024-01-05

**Authors:** Jialu Li, Yiwei Hao, Ying Liu, Liang Wu, Hongyuan Liang, Liang Ni, Fang Wang, Sa Wang, Yujiao Duan, Qiuhua Xu, Jinjing Xiao, Di Yang, Guiju Gao, Yi Ding, Chengyu Gao, Jiang Xiao, Hongxin Zhao

**Affiliations:** ^1^Clinical and Research Center of AIDS, Beijing Ditan Hospital, Capital Medical University, Beijing, China; ^2^Division of Medical Record and Statistics, Beijing Ditan Hospital, Capital Medical University, Beijing, China; ^3^Department of Clinical Medicine, Zhengzhou University, Zhengzhou, China

**Keywords:** HIV, AIDS, machine learning, length of stay, risk factors, calibration curves

## Abstract

**Objective:**

The study aimed to use supervised machine learning models to predict the length and risk of prolonged hospitalization in PLWHs to help physicians timely clinical intervention and avoid waste of health resources.

**Methods:**

Regression models were established based on RF, KNN, SVM, and XGB to predict the length of hospital stay using RMSE, MAE, MAPE, and *R*^2^, while classification models were established based on RF, KNN, SVM, NN, and XGB to predict risk of prolonged hospital stay using accuracy, PPV, NPV, specificity, sensitivity, and kappa, and visualization evaluation based on AUROC, AUPRC, calibration curves and decision curves of all models were used for internally validation.

**Results:**

In regression models, XGB model performed best in the internal validation (RMSE = 16.81, MAE = 10.39, MAPE = 0.98, *R*^2^ = 0.47) to predict the length of hospital stay, while in classification models, NN model presented good fitting and stable features and performed best in testing sets, with excellent accuracy (0.7623), PPV (0.7853), NPV (0.7092), sensitivity (0.8754), specificity (0.5882), and kappa (0.4672), and further visualization evaluation indicated that the largest AUROC (0.9779), AUPRC (0.773) and well-performed calibration curve and decision curve in the internal validation.

**Conclusion:**

This study showed that XGB model was effective in predicting the length of hospital stay, while NN model was effective in predicting the risk of prolonged hospitalization in PLWH. Based on predictive models, an intelligent medical prediction system may be developed to effectively predict the length of stay and risk of HIV patients according to their medical records, which helped reduce the waste of healthcare resources.

## Introduction

According to estimates from UNAIDS (https://www.unaids.org/en) (1), there are 38.4 million people living with HIV (PLWH), and 1.5 million new PLWH were diagnosed and 650,000 were dead all over the world by 2021. In recent years, with the widespread use of antiretroviral therapy (ART), the lifespan prolonged and mortality decreased significantly (2, 3). Although survival status had improved among PLWH, HIV-associated comorbidity, including opportunistic infections (4), acquired immune deficiency syndrome (AIDS)-defining cancers and non-AIDS-defining events (NADEs) (5), remained major problems and posed a great challenge to survival quality among PLWH in China.

Although HIV infection became a chronic disease, opportunistic infections (4–6) or AIDS-defining cancers were diagnosed in some PLWHs due to unware of HIV infection or ART failure, while NADEs gradually occurred in PLWHs receiving ART, including cardiovascular diseases, metabolic disorders, hepatic and renal diseases. The diagnosis and management of these HIV-associated comorbidity placed a significant burden on healthcare resources. Indeed, a large portion of health economic burden associated with HIV was attributed to the cost of hospital care and treatment for PLWHs (7–9).

Due to dramatic increases in healthcare costs and admission expenditures, accurate prediction of length of hospital stay and identify the risk factors of prolonged hospital stay helped physicians plan interventions in diagnosis and management for PLWHs with HIV-associated comorbidity, which was important to reduce waste of hospital resources (10, 11).

Machine learning (ML) algorithms could build complex nonlinear predictive models, which connected independent features with the relevant risk factors in large data sets and presented highly efficient and accurate characteristics (12, 13). In recent decades, although ML algorithm was widely accepted and applied to medical and healthcare problems in establishing predictive models, especially in the field of oncology (14) and intensive care medicine (15), little was done to develop predictive models in the field of HIV/AIDS in China. The aim of this study was to use multiple ML predictive models to predict length of hospital stay and assess the risk of prolonged hospital stay among PLWHs, which helped establish an intelligent medical diagnosis and management system and helped physicians timely clinical intervention and avoid waste of health resources.

## Methods

### Ethical consideration

This observational study was carried out in Beijing Ditan Hospital, Capital Medical University, the largest referral hospital of HIV/AIDS in China, and all procedures in this study was approved by the Human Science and Ethics Committee of Beijing Ditan Hospital, which agreed to waive requirement for informed consent based on characteristics of observational and retrospective study.

### Study design

**Figure 1:**
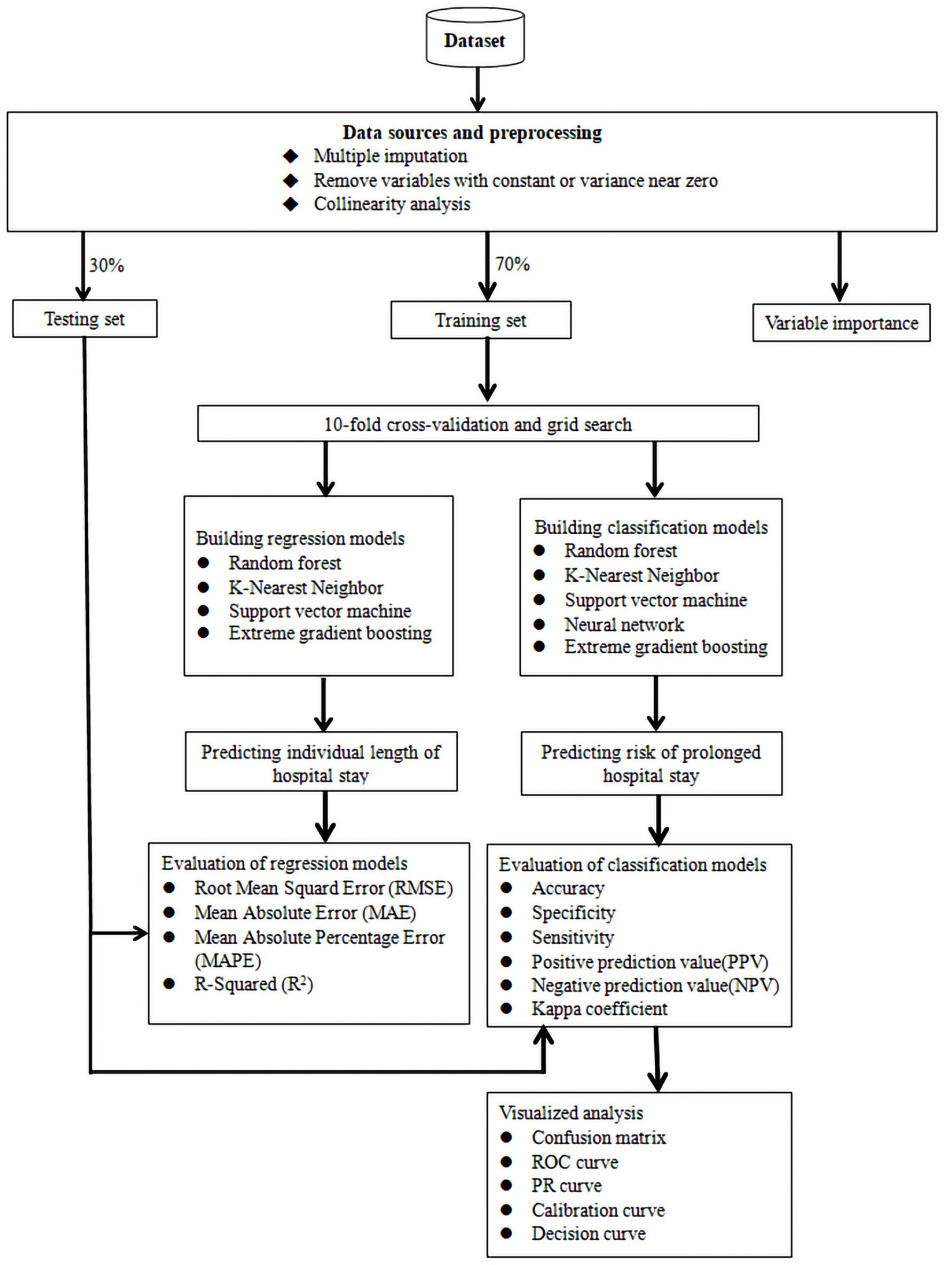
Study flow diagram of data preparation and model prediction. ROC, receiver operating characteristic curve; PR, precision-recall.

A cohort of inpatients was included in the study, who were enrolled due to different AIDS-defining illnesses and NADEs at Beijing Ditan Hospital, Capital Medical University, Beijing, from January, 2008 to June, 2020. After clinical data was collected and preprocessed, variable importance was evaluated and study subjects was divided randomly into training and testing sets, and we established two models based on methodology of machine learning (ML): (1) a model to predict the risk of prolonged hospital stay, which was evaluated with accuracy, specificity, sensitivity, positive and negative predictive value and kappa coefficient, and received visualization analysis based on confusion matrix, ROC curve, PR curve, calibration curve and decision curve; and (2)another model to predict individual length of hospital stay, which was evaluated with root mean squard error (RMSE), mean absolute error (MAE), mean absolute percentage error (MAPE) and R-squared (*R*^2^). We validated these 2 kinds of models in the testing set to perform internal validation to determine the optimal model (Figure 1).

### Data sources

Supplementary Table S1 presented that the study variables were polytomous variables, including age, marital status, route of transmission and HAART while other variables as binary variables. All admitted patients over eighteen years old were included in this study, while patients admitted for <12 h were excluded due to incomplete clinical data and more than 30% variable missing in the study (Supplementary Table S1). Raw clinical data were extracted from the hospital electronic medical records. Demographic data included age, gender, marital status, and clinical data included route of HIV transmission, type of admission, baseline HAART at current admission, baseline CD4 cell count, baseline viral load, admission to the intensive care unit (ICU) and final diagnosis, including different opportunistic infections and NADEs (Supplementary Table S2).

### Outcomes

The primary outcome of interest was the numeric length of hospital stay (duration between the admission and discharge).

The prolonged hospital stay was defined as more than 25 days between the admission and discharge based on literature reports (16), and the secondary outcome of interest was the risk of prolonged hospital stay.

### Data pre-processing and preparation

Data pre-processing and preparation were conducted among the included study subjects, whose missing variables were filled in Supplementary Table S1 based on the Multiple Imputation by Chained Equations (MICE) algorithm (17, 18).

After preprocessing of clinical data of study subjects, the random sampling was carried out using R software (version 4.2.1), 70% of study subjects were randomly sampled and included the training set, while another 30% as the testing set, which provided unbiased model validity.

### Definition

HIV-infected population had increased risk for non-AIDS-defining events (NADEs) (19), which included cardiovascular and cerebrovascular diseases, metabolic diseases, renal diseases, liver diseases, osteoporosis, and non-AIDS-defining cancers.

Unexplained infections were diagnosed based on routine and biochemical tests of opportunistic pathogens with different samples, but no definite pathogens were found (20).

Multiple opportunistic infections (OIs) were defined that two or more opportunistic pathogens were definitely diagnosed and co-existed *in vivo*, which were used into respiratory system or central neural system (20).

Tuberculosis disseminated in multiple organs meant tuberculosis was diagnosed in multiple organs, including lung, lymph nodes, and central neural system.

The diagnosis and treatment of AIDS-defining illnesses, including OIs and opportunistic malignancies, was carried out based on *Guidelines for Prevention and Treatment of Opportunistic Infections in HIV-Infected Adults and Adolescents* recommended by the U.S. Centers for Disease Control and Prevention (CDC) (21), while the diagnosis and management of NADEs was conducted on the basis of HIVBOOK (22).

### Variable importance

Variable importance was evaluated prior to running all models based on extreme gradient boosting (XGB) model due to a form of gradient boosting without over-fitting (23), which was prone to create a suitable model based on the evaluation of variable (24).

### Predicting individual length of hospital stay based on ML regression model

For the training set, 10-fold cross validation and grid search was used to obtain the best model hyper-parameters, which were used into four ML regression model algorithms, including random forest (RF), k-Nearest Neighbor (KNN), support vector machine (SVM), and extreme gradient boosting (XGB) (25, 26) to predict the length of hospital stay in PLWHs. The root mean squard error (RMSE), mean absolute error (MAE), mean absolute percentage error (MAPE) and R-squared (*R*^2^) were calculated respectively to evaluate the performance of different regression models.

Internally validation of machine learning (ML) predictive models was performed in the testing set to determine the optimal model for assessing the risk of prolonged hospital stay and of the length of hospital stay (LOS).

### Predicting risk of prolonged hospital stay based on ML classification model

In the training set, 10-fold cross validation and grid search were used to obtain the best model hyper-parameters, which were used in five ML regression model algorithms, including RF, KNN, SVM, NN, and XGB, to predict the risk of prolonged hospital stay beyond 25 days (16).

The receiver operating characteristic curve (ROC) and the precision recall curve (PRC) for the training set were plotted respectively and the area under the receiver operating characteristic curves and the precision recall curve (AUROC and AUPRC) were calculated to evaluate the performance of different classification models. We further evaluated other performance metrics, including accuracy, specificity, sensitivity, positive prediction value (PPV), negative prediction value (NPV), and kappa coefficient of different classification models.

The evaluation of the visualization was based on calibration curves, decision curves analysis (DCA) and confusion matrices (27, 28).

We validated predictive classification models in the testing set to determine the optimal model for internal validation for evaluation of the risk of prolonged hospital stay.

### Statistical analysis

The statistical analysis in this study was performed using R version 4.2.1 (packages caret, yardstick, modEvA and runway), and the application codes and models were publicly available at Github (https://github.com/igor-peres).

## Result

Based on the inclusion and exclusion criteria, 1,556 inpatients were included in the study at Beijing Ditan Hospital, Capital Medical University from January, 2008 to June, 2020. 1418 cases (91.1%) were male and 138 cases (8.9%) were female, and average age was 45 years among study subjects, in which average baseline CD4 cell counts was 158 cells/ul, and systemic multiple OIs were diagnosed in 779 cases (50.1%) while NADEs were found in 51 cases (3.3%). The average length of hospital stay was 24.14 days among these study subjects, and demographic and clinical characteristics was detailed in Supplementary Table S2.

### Data preparation and preprocessing

In this study, data were prepared and no variable was missing by more than 30%, which were re-evaluated based on MICE algorithm (Supplementary Table S1) (17, 18). More than 25 days was regarded as prolonged hospital stay based on literature reports (16), which corresponded to 36% of study subjects (564 ones) in this study.

Random assignment in 7:3 ratio was carried out among study subjects to form training cohort (*n* = 1089) and validation cohort (*n* = 467) for predicting the length of hospital stay based on ML regression model and predicting the risk of prolonged hospital stay based on ML classification model.

### The evaluation of variable importance

**Figure 2:**
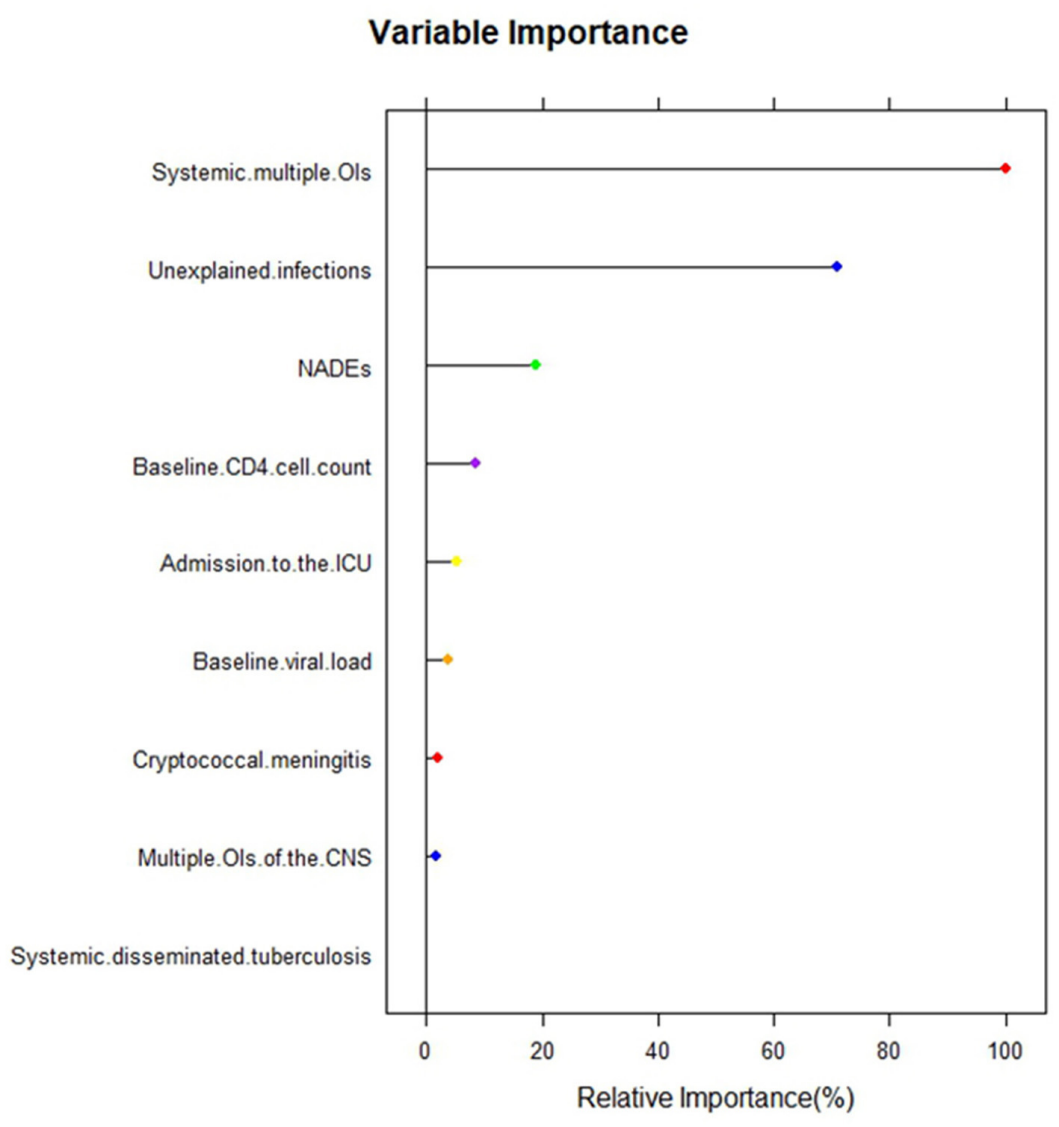
The importance of features in the extreme gradient boosting (XGB) model. CNS, central nervous system; ICU, intensive care unit; Ois, opportunistic infections; NADEs, non-aids-defining events.

The extreme gradient boosting (XGB) model was selected from multiple machine learning models based on previous report (24) and used to demonstrate the importance of the included features that contributed to the prolonged hospital stay (Supplementary Table S3). Systemic multiple OIs was the most important variable, followed by unexplained infections, NADEs, baseline CD4 cell count, admission to the ICU, baseline viral load, cryptococcal meningitis, multiple OIs in the central nervous system (CNS), and systemic disseminated tuberculosis (Figure 2).

### Predicting the length of hospital stay based on ML regression model

Four different ML regression models were run to predict the individual length of hospital stay among PLWHs, which was described in Table 1. In the training set, KNN model obtained the best discriminative capability (RMSE = 12.72, MAE = 7.23, MAPE = 0.60, *R*^2^ = 0.68; Table 1).

**Table 1:** Statistical comparison based on Machine Learning Regression Models to predict individual length of hospital stay.

	**Models**	**RMSE**	**MAE**	**MAPE**	***R* ^2^**
Training set	RF	14.67	8.67	0.84	0.58
	KNN	12.72	7.23	0.60	0.68
	SVM	16.83	8.25	0.63	0.44
	XGB	17.11	9.84	0.86	0.42
Testing set	RF	18.21	10.91	1.11	0.37
	KNN	19.67	11.61	0.99	0.27
	SVM	18.05	10.36	0.85	0.39
	XGB	16.81	10.39	0.98	0.47

RF, random forest; SVM, support vector machine; KNN, k-nearest neighbor; XGB, extreme gradient boosting; RMSE, root mean square error; MAE, mean absolute error; MAPE, mean absolute percentage error; R^2^ (R-Squared), coefficient of determination.

The internal validation was further carried out in the testing set, and found that the XGB model performed best (RMSE = 16.81, MAE = 10.39, MAPE = 0.98, *R*^2^ = 0.47), followed by SVM model (RMSE = 18.05, MAE = 10.36, MAPE = 0.85, *R*^2^ = 0.39), RF model (RMSE = 18.21, MAE = 10.91, MAPE = 1.11, *R*^2^ = 0.37), and KNN model (RMSE = 19.67, MAE = 11.61, MAPE = 0.99, *R*^2^ = 0.27; Table 1).

Based on the evaluation of different models, including RF, KNN, SVM, and XGB in the training and testing sets, the error and *R*^2^ of the XGB model changed slightly and performed stable (Table 1), which indicated that XGB model presented a better fitting and more stable and effective in predicting the length of stay than RF, KNN, and SVM.

### Predicting the risk of prolonged hospital stay based on ML classification model

**Figure 3:**
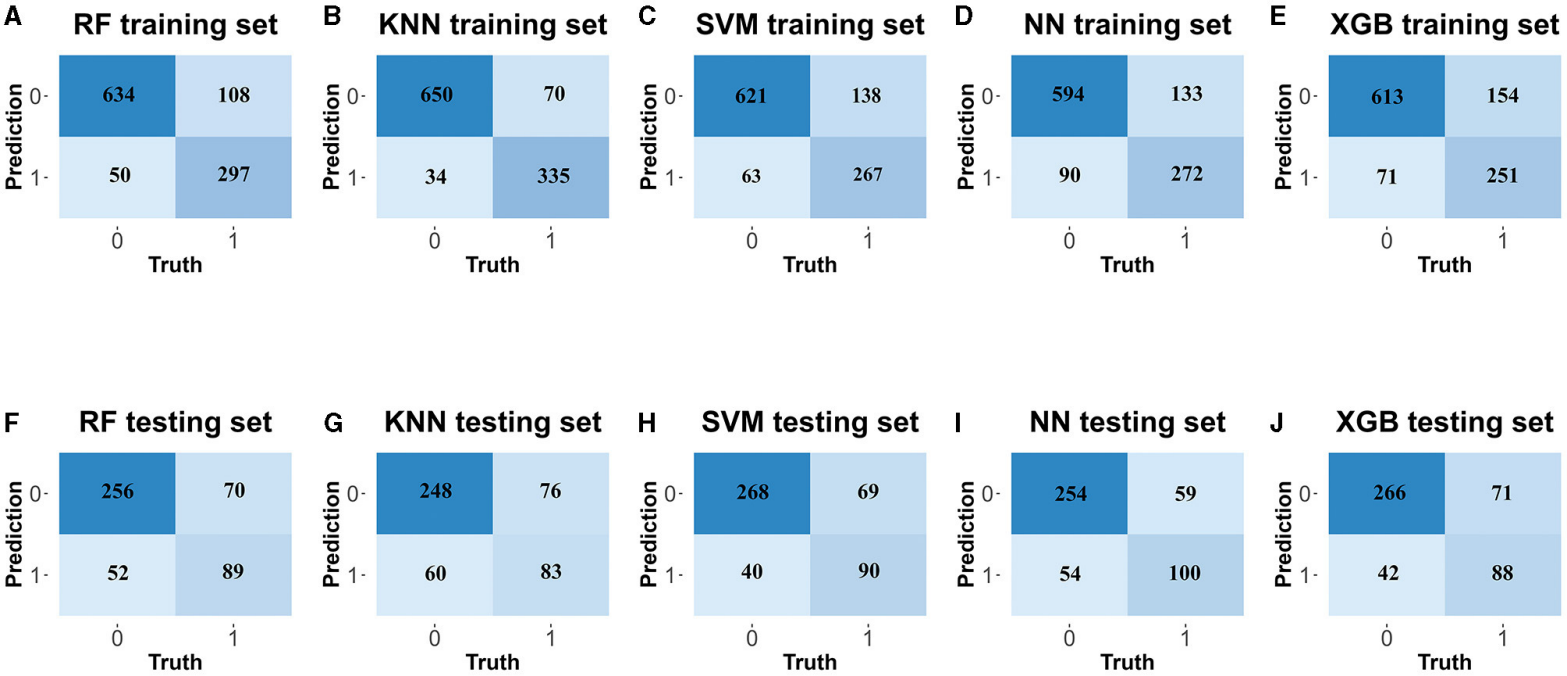
The confusion matrix of classification machine learning models. In the training set: **(A)** the random forest (RF), **(C)** k-Nearest Neighbor (KNN), **(E)** support vector machine (SVM), **(G)** neural network (NN), **(I)** extreme gradient boosting (XGB). In the testing set: **(B)** the random forest (RF), **(D)** k-Nearest Neighbor (KNN), **(F)** support vector machine (SVM), **(H)** neural network (NN), **(J)** extreme gradient boosting (XGB).

Five different ML classification models were run to predict the risk of prolonged hospital stay among PLWHs (Table 2). In the training set, KNN model obtained the best discriminative capability (accuracy = 0.9008, PPV = 0.8982, NPV = 0.9063, sensitivity = 0.9525, specificity = 0.8096, Kappa = 0.7802; Table 2, Figure 3).

**Table 2:** Performance indicators of confusion matrix in training and testing sets based on classification model machine learning algorithms.

**Model**	**Training set**						**Testing set**						
	**Accuracy (95% CI)**	**PPV**	**NPV**	**Sensitivity**	**Specificity**	**Kappa**	**Accuracy (95% CI)**	**PPV**	**NPV**	**Sensitivity**	**Specificity**	**Kappa**	**Kappa**
RF	0.8567 (0.8345, 0.8770)	0.8505	0.8719	0.9410	0.7081	0.6766	0.7473 (0.7053, 0.7861)	0.7720	0.6884	0.8552	0.5588	0.4314	0.4344
KNN	0.9008 (0.8815, 0.9179)	0.8982	0.9063	0.9525	0.8096	0.7802	0.7281 (0.6853, 0.7679)	0.7607	0.6525	0.8350	0.5412	0.3904	0.3519
SVM	0.8154 (0.7911, 0.8381)	0.8208	0.8025	0.9094	0.6497	0.6382	0.7473 (0.7053, 0.7861)	0.7737	0.6857	0.8519	0.5647	0.4329	0.4225
NN	0.7961 (0.7710, 0.8197)	0.8124	0.7590	0.8849	0.6396	0.5430	0.7623 (0.7210, 0.8002)	0.7853	0.7092	0.8620	0.5882	0.4672	0.4023
XGB	0.7971 (0.7719, 0.8206)	0.8118	0.7629	0.8878	0.6371	0.5443	0.7666 (0.7255, 0.8042)	0.7831	0.7259	0.8754	0.5765	0.4727	

RF, random forest; SVM, support vector machine; KNN, k-nearest neighbor; NN, neural network; XGB, extreme gradient boosting; PPV, positive prediction value; NPV, negative prediction value.

In the testing set, the further evaluation presented as following: the NN model (accuracy = 0.7623, PPV = 0.7853, NPV = 0.7092, sensitivity = 0.8620, specificity = 0.5882, Kappa = 0.4672), XGB model (accuracy = 0.7666, PPV = 0.7831, NPV = 0.7259, sensitivity = 0.8754, specificity = 0.5765, Kappa = 0.4727), SVM model (accuracy = 0.7473, PPV = 0.7737, NPV = 0.6857, sensitivity = 0.8519, specificity = 0.5647, Kappa = 0.4329), RF model (accuracy = 0.7473, PPV = 0.7720, NPV = 0.6884, sensitivity = 0.8552, specificity = 0.5588, Kappa = 0.4314), and KNN model (accuracy = 0.7281, PPV = 0.7607, NPV = 0.6525, sensitivity = 0.8350, specificity = 0.5647, Kappa = 0.3904; Table 2, Figure 3).

Based on the evaluation of different models, including RF, KNN, SVM, NN, and XGB in the training and testing sets, the accuracy, PPV, NPV, sensitivity, specificity and kappa efficiency of the NN model changed slightly and performed stable (Table 2), which indicated that NN model presented a better fitting and more effective in predicting the risk of prolonged hospital stay than RF, KNN, SVM, and XGB.

### Visualization evaluation of ML classification models to predict the risk of prolonged hospital stay

**Figure 4:**
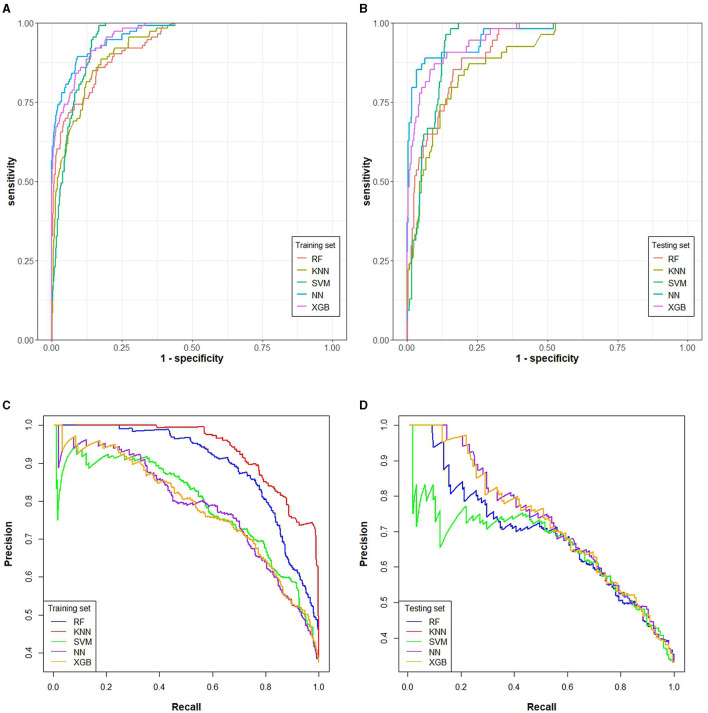
The area under ROC and PR of five classification machine learning models. RF, KNN, SVM, NN, and XGB algorithms were included in all ROC and PR curves. **(A)** AUROC of the training set, **(B)** AUPRC of the training set, **(C)** AUROC of the testing set, **(D)** AUPRC of the testing set.

In addition, AUROC of all five models exceeded 0.9 in both the training and testing sets. Compared to RF, KNN, SVM, and XGB in the training set (AUROC_RF_ = 0.9315, AUROC_KNN_ = 0.9305, AUROC_SVM_ = 0.9518, AUROC_XGB_ = 0.9674, Figure 4, Supplementary Table S4), the NN model had the largest area under the ROC curve (AUROC_NN_ = 0.9739, Figure 4, Supplementary Table S4). Similarly, in the testing set, compared to the RF, KNN, SVM, and XGB (AUROC_RF_ = 0.9315, AUROC_KNN_ = 0.9225, AUROC_SVM =_ 0.9419, AUROC_XGB_ = 0.9695, Figure 4, Supplementary Table S4), the NN model also presented the largest area under the ROC curve (AUROC_NN_ = 0.9779, Figure 4, Supplementary Table S4).

The evaluation of PR curves indicated that, in the training set, the AUPRC of RF, KNN, SVM, NN, and XGB models was 0.896, 0.862, 0.691, 0.765, and 0.678, respectively (Figure 4, Supplementary Table S4), while in the testing set, the AUPRC of RF, KNN, SVM, NN, and XGB models was 0.755, 0.643, 0.679, 0.773, and 0.712, respectively (Figure 4, Supplementary Table S4) in the testing set. Compared with the PR curves of the training set, the AUPRC of all models decreased in the testing set, while the AUPRC of the NN model changed slightly and performed stable, which indicated it was an optimal ML model.

At the same time, the calibration curves (Supplementary Figure S1) indicated that all models including NN model had good predictive ability, while NN model also presented the best predictive value based on the decision curves analysis (Figure 2).

## Discussion

Despite of availability of antiretroviral therapy among PLWHs, opportunistic infections and NADEs were the main reason of hospitalization in China, and individual and social healthcare burden was increased due to medical cost and prolonged hospital stay, which indicated that an intelligent medical system based on ML models were required to predict individual length of hospital stay and risk factors of prolonged hospital stay. In addition, the intelligent medical system based on ML models could help improve diagnosis, treatment and care delivery, reduce medical cost, identify the individuals with prolonged hospital stay in time and establish preventive strategies to reduce the cost and shorten the length of hospital stay.

Previous studies have elucidated the risk factors of prolonged hospitalization among PLWHs (29), while few studies have predicted individual length of hospital stay among PLWHs (30). Coelho et al. (31) studied 30-day readmission rates in a HIV-infected cohort in Brazil, and found risk factors for readmission, which contributed to prognosis and early follow-up after discharge. The above conclusions were made based on PLWHs in foreign countries, while rarely studies were conducted to predict length of hospital stay and risk factors of prolonged hospital stay among HIV/AIDS population in China. In this study, we studied length of hospital stay and risk factors of prolonged hospital stay based on ML models for the first time among PLWHs in China, and we found that systemic multiple OIs, unexplained infections, NADEs, baseline CD4 cell count, admission to the ICU, baseline viral load, cryptococcal meningitis, multiple OIs of the CNS and systemic disseminated tuberculosis were significantly associated with prolonged hospital stay, which can be used to develop strategies for diagnosis, treatment and prophylaxis for HIV-associated OIs and NADEs and shortening length of hospital stay and reduced medical cost.

Sensitivity analysis had important applications in model calibration, where focusing on sensitive parameters could be used to simplify the calibration phase and improved model performance in calibrated models with large parameters. Hyper-parameters had a considerable impact on the model performance in ML, and optimization of hyper-parameters of ML models was needed to improve their performance (32). The improper selection of hyper-parameters can significantly affect the prediction model results, and 10-fold cross-validation and grid search were recommended to use to achieve the optimal hyper-parameters in the training set (33). In this study, the original set was randomly divided into training set for establishing predictive models and testing set for validating models based on stratified sampling (34). Ten-fold cross-validation and grid search were performed to reduce model over-fitting and obtain superior parameters based on relevant literature recommendation.

In this study, several ML models were established separately to predict the length of hospital stay and the risk of prolonged hospitalization in people living with HIV/AIDS. Better performance of the metrics and smaller differences in metrics between the training and test sets represents a more stable and better model, in which XGB performed best in prediction of individual length of hospital stay while NN model was the optimal one for predicting the risk of prolonged hospitalization in this study.

ML models can be applied to predict the length of hospitalization for diseases. Extreme Gradient Boosting (XGB) Algorithm is a machine learning one which can improve the integration of multiple decision trees by gradient boosting method, which is characterized by high accuracy, difficulty of adaptation and good scalability. Chen et al. (24) indicated that the XGB algorithm was a suitable ML model for predicting length of hospital stay in ischemic stroke patients with high accuracy, less over-fitting and scalability and Grovu et al. (35) showed that the XGB model has higher accuracy and better performance in predicting the length of hospitalization compared with other algorithms. Morgan et al. (36) indicted that the accuracy and stability of ML models was improved and presented less over-fitting as the sample size increased and a more stable ML model in training and testing sets was necessary to be selected in clinical practice. In this study, we found that the best models for the training and testing sets were different due to relative small sample size, the RF, KNN, and SVM models presented increased error and decreased *R*^2^ values between training and testing sets, which indicated unstable and over-fitting models, while XGB presented stable and excellent fitting features between training and testing sets, which indicated that XGB was an optimal ML model to predict individuals length of hospital stay among HIV-infected population.

The risk factors of prolonged hospital stay were evaluated based on ML classification model, and we found that the accuracy of all models was higher than 70% and the AUROC was more than 0.9, indicating that the ML models were feasible tools to predict risk of prolonged hospital stay. Ahlstrom et al. (37) also reported ML models were applied to predict HIV status, which was similar with our conclusion that ML models could be used in clinical prediction. In this study, we used the ML models to predict the risk of prolonged hospital stay in HIV-infected individuals in China, and we found that NN model presented best performance and less over-fitting in predicting risk of hospital stay. Neural networks were a ML modeling method that used data obtained from previous experiments to adapt to new situations or to control unknown systems, and could be considered as a tool for molecular data analysis and interpretation (38). Kulkarni et al. (39) indicated that neural networks could be used to predict l prolonged length of hospital stay in clinical work. Van der Ploeg et al. (40) indicated that modern models including SVM, NN, and RF may require more than 10 times the number of study subjects per variable to achieve stable models, and for small samples of data, any models may be prone to perform unstable. We found that the evaluation of KNN and RF models presented unstable features, in which accuracy, sensitivity, specificity, PPV and NPV, and kappa decreased significantly between training and testing sets, while NN and XGB presented stable and excellent fitting features between training and testing sets, further analysis indicated that the indicators of NN model performed better than XGB model, which indicated that NN model was an optimal ML model to predict risk of hospital stay among HIV-infected population.

The evaluation of visualization indicated that the NN model performed better in both training and testing sets in terms of AUROC, AUPRC, calibration curves and decision curves. The predicted values of the calibration curves of all models presented similar results with the true values, indicating that these models, including NN ones, performed well based on calibration curves. For the decision curves, the NN model had the larger net gain than other models after intervention. Comprehensive assessment, instead of using only one indicator such as the AUC (41, 42), was also used in this study, which helped find the optimal model, NN model, for prolonged hospital stay (43).

Based on ML model prediction, medical and nursing service may be reduced by identifying individuals at risk for prolonged stay at the time of admission or hospitalization, assigning dedicated physicians, and conducting schedule of reasonable discharge after a continuum of care. The medical cost and insurance that was often considered as important socioeconomic factors also significantly affected the length of hospital stay (44, 45), and the predictive models in this study helped individuals' stratification based on risk factors of prolonged hospital stay, and reduce excessive waste of healthcare resources (9).

This study has some advantage. First, we used ML regression and classification models to predict individual length of hospital stay and risk factors of prolonged hospital stay, which helped adequately evaluate length of hospital stay among PLWHs. Second, several different ML algorithms were used and internal validation was performed in these two kinds of models, which indicated good accuracy and credibility in these predictive models. Third, 12-year clinical data were used to establish predictive models, based on stable spectrum of HIV-associated diseases (46), which indicated its reliability.

This study had several limitations. First, this study was a retrospective study and potential selection bias and information bias was inevitable (47–49). Second, this was a single-center study so that our findings may not be generalizable to other hospitals where discharge criteria may differ due to difference in understanding of HIV/AIDS in different hospital, but not referral pattern bias. Third, the individual's social environment such as social discrimination and rehabilitation care after discharge could easily influenced the prognosis and admission. In addition, external validation was not conducted in this study. Some of the established models have performed well in internal validation, but it is necessary to validate their generalizability in separate study to further update the models.

In this study, we found that the ML models, based on existing technology, medicine, and specialized wards to treat patients, may play a meaningful role in predicting length of hospital stay and the risk factors of prolonged stay among PLWHs in Beijing Ditan Hospital, Capital Medical University. These predictive models may help healthcare workers determine the likelihood and risk of prolonged length of hospital stay among PLWHs to adjust strategies of diagnosis, treatment and care delivery. Understanding the main influencing factors about individual hospital stay based on the predictive model will allow us to contribute to the scheduling of reasonable discharges and early recovery for PLWHs.

## Conclusion

In conclusion, the study aimed to adopt various machine learning techniques to predict the length of hospital stay and risk of prolonged hospitalization in PLWHs. The results indicated that XGB model was effective in predicting length of hospital stay of PLWHs, while NN model was effective in predicting the risk of prolonged hospitalization of PLWHs with high accuracy, which indicated well-performing calibration curve and decision curve. Our study also identified important features that could be used for these models, including systemic multiple OIs, unexplained infections, NADEs, baseline CD4 cell count, admission to the ICU, baseline viral load, cryptococcal meningitis, multiple OIs of the CNS and systemic disseminated tuberculosis. Based on predictive models, an intelligent medical prediction system may be developed to effectively predict the duration and risk of prolonged length of stay in PLWHs according to their medical records to help reduce the waste of healthcare resources.

## Data availability statement

The original contributions presented in the study are included in the article/Supplementary material, further inquiries can be directed to the corresponding authors.

## Ethics statement

The studies involving humans were approved by Human Science and Ethics Committee of Beijing Ditan Hospital. The studies were conducted in accordance with the local legislation and institutional requirements. The Ethics Committee/institutional review board waived the requirement of written informed consent for participation from the participants or the participants' legal guardians/next of kin based on the characteristics of the observational and retrospective study.

## Author contributions

JL: Formal analysis, Conceptualization, Data curation, Investigation, Methodology, Software, Writing – original draft, Writing – review & editing. YH: Conceptualization, Data curation, Investigation, Methodology, Formal analysis, Writing – review & editing. YL: Data curation, Formal analysis, Methodology, Software, Writing – review & editing. LW: Data curation, Investigation, Methodology, Supervision, Writing – review & editing. HL: Data curation, Investigation, Software, Writing – review & editing. LN: Data curation, Investigation, Software, Writing – review & editing. FW: Data curation, Methodology, Software, Supervision, Writing – review & editing. SW: Data curation, Investigation, Methodology, Software, Writing – review & editing. YD: Data curation, Investigation, Methodology, Software, Writing – review & editing. QX: Data curation, Investigation, Methodology, Software, Writing – review & editing. JinX: Data curation, Investigation, Methodology, Software, Writing – review & editing. DY: Data curation, Investigation, Methodology, Software, Writing – review & editing. GG: Conceptualization, Investigation, Methodology, Software, Supervision, Writing – review & editing. YD: Data curation, Investigation, Methodology, Software, Writing – review & editing. CG: Data curation, Investigation, Methodology, Software, Writing – review & editing. JaiX: Formal analysis, Funding acquisition, Project administration, Resources, Supervision, Validation, Visualization, Writing – review & editing. HZ: Formal analysis, Funding acquisition, Project administration, Resources, Supervision, Validation, Visualization, Writing – review & editing.

## Supplementary material

Supplementary Figure S1 Calibration curves for five classification ML models in internal validation (RF, KNN, SVM, NN, and XGB) to predict risk of prolonged hospital stay.

Supplementary Figure S2 Decision curves for five classification ML models in internal validation (RF, KNN, SVM, NN, and XGB) to predict the risk of prolonged hospital stay.
