# Supplementary material for: Supervised machine learning algorithms to predict the duration and risk of long-term hospitalization in HIV-infected individuals: a retrospective study
Source: Front Public Health. 2024 Jan 5;11:1282324. doi: 10.3389/fpubh.2023.1282324 (PMC10796994; doi:10.3389/fpubh.2023.1282324)
Supplement: Supplementary file 1 [file Table_1.docx]

**Table S1: Data Dictionary for the final dataset after the inclusion criteria**

|  | **Features** | **Description** | **Type** | **Missing(%)** |
| --- | --- | --- | --- | --- |
| Admission | Age | Age（0:18-34 years old,1:35-49 years old,2:≥50 years old） | Polytomous | 0% |
| Admission | Gender | Gender（1:male ,2:female） | Binary | 0% |
| Admission | Marital status | Marital.status (1: Single ,2: Married or cohabiting, 3: Divorced or separation, 4: Widowed, 5: Unknown) | Polytomous | 0.1% |
| Admission | Route of HIV transmission | Route of virus infection (1.Same-sex transmission, 2.Heterosexual transmission ,3. Blood transfusion, 4. Drug abuse ,5. Unknown) | Polytomous | 0% |
| Admission | Baseline CD4 cell count | CD4 cell count at admission(200 is the boundary) | Binary | 6% |
| Admission | Baseline viral load | Viral load on admission(200,000 copies is the boundary) | Binary | 29% |
| Admission | Type of admission | Type of hospital admission (1:Outpatient, 2:Emergency) | Binary | 13% |
| Admission | HAART | ART therapy(naive or less than or at least 6 months) | Polytomous | 0% |
| Diagnosis | Admission to the ICU | Whether the patients stay in the ICU (yes or no) | Binary | 0% |
| Diagnosis | Unexplained infections | Type of hospital admission (yes or no) | Binary | 0% |
| Diagnosis | PCP | Main diagnosis of admission (yes or no) | Binary | 0% |
| Diagnosis | Invasive fungal infections | Main diagnosis of admission (yes or no) | Binary | 0% |
| Diagnosis | Cytomegalovirus infections(CMV) | Main diagnosis of admission (yes or no) | Binary | 0% |
| Diagnosis | Syphilis | Main diagnosis of admission (yes or no) | Binary | 0% |
| Diagnosis | Pulmonary tuberculosis | Main diagnosis of admission (yes or no) | Binary | 0% |
| Diagnosis | Cryptococcal meningitis | Main diagnosis of admission (yes or no) | Binary | 0% |
| Diagnosis | Mycobacterium avium complex infections(MAC) | Main diagnosis of admission (yes or no) | Binary | 0% |
| Diagnosis | Non-aids-defining events | Including cardiovascular and cerebrovascular diseases, metabolic diseases, renal diseases, liver diseases, osteoporosis and non-AIDS-defining cancers (yes or no) | Binary | 0% |
| Diagnosis | Systemic multiple opportunistic infections(OIs) | More than two pathogenic microorganisms infect the human body(yes or no) | Binary | 0% |
| Diagnosis | Multiple opportunistic infections (OIs) of the CNS | More than two pathogenic microorganisms infect the human central nervous system(yes or no) | Binary | 0% |
| Diagnosis | Multiple opportunistic infections (OIs) in respiratory system | More than two pathogenic microorganisms infect the human central nervous system(yes or no) | Binary | 0% |
| Diagnosis | Systemic disseminated tuberculosis | Tuberculosis infection in multiple organs(yes or no) | Binary | 0% |

Note: CNS:Central nervous system, HAART: highly active anti-retroviral therapy, ICU: intensive care unit, MAC: mycobacterium avium complex, OIs:opportunistic infections, PCP: Pneumocystis carinii pneumonia.
