# Supplementary material for: Supervised machine learning algorithms to predict the duration and risk of long-term hospitalization in HIV-infected individuals: a retrospective study
Source: Front Public Health. 2024 Jan 5;11:1282324. doi: 10.3389/fpubh.2023.1282324 (PMC10796994; doi:10.3389/fpubh.2023.1282324)
Supplement: Supplementary file 2 [file Table_2.docx]

**Table S2：Baseline clinical characteristics of study subjects (n=1556)**

|  | **Total** | **LOS < 25 days** | **LOS >= 25 days** | **p value** |
| --- | --- | --- | --- | --- |
| Age, years ( IQR) | 45.12 (36.00,52.00) | 45.13(36.00,52.00) | 45.09 (36.00,52.00) | 0.710 |
| Gender,n(%) |  |  |  | 0.094 |
| male | 1418 (91.1) | 895 (90.2) | 523 (92.7) |  |
| female | 138（8.9） | 97（9.8） | 41（7.3） |  |
| Marital status,n(%) |  |  |  | 0.436 |
| Single | 904（58.1） | 568（57.4） | 336（59.6） |  |
| Married or cohabiting | 518（33.2） | 332（33.6） | 186（33.0） |  |
| Divorced or separation | 118（7.6） | 78（7.9） | 40（7.0） |  |
| Widowed | 12（0.8） | 10（1.0） | 2（0.4） |  |
| Unknown | 4（0.3） | 4（0.1） | 0（0） |  |
| Route of HIV transmission, n(%) |  |  |  | 0.040 |
| Same-sex transmission | 1025（65.9） | 669（67.4） | 356（63.1） |  |
| Heterosexual transmission | 265（17.0） | 175（17.6） | 90（16.0） |  |
| Blood transfusion | 34（2.2） | 21（2.1） | 13（2.3） |  |
| Drug abuse | 13（0.8） | 6（0.6） | 7（1.2） |  |
| Unknown | 219（14.1） | 121（12.2） | 98（17.4） |  |
| Baseline CD4 cell count , /ul (IQR) | 157.81 (24.00,230.00) | 159.21 (25.00 ,230.75) | 155.34 (23.00,229.75) | 0.639 |
| Baseline viral load, copies/ml(IQR) | 359590.97 (75234.25, 359591.00) | 370248.60 (74728.25, 359591.00) | 340845.65 (75658.50, 359591.00) | 0.910 |
| Type of admission ,n(%) |  |  |  | 0.590 |
| Outpatient | 1272（81.7） | 807（81.4） | 465（82.4） |  |
| Emergency | 284（18.3） | 185（18.6） | 99（17.6） |  |
| HAART ,n(%) |  |  |  | 0.001 |
| Naive | 987（63.4） | 537（54.1） | 450（79.8） |  |
| Less than 6 months | 251（16.1） | 186（18.8） | 65（11.5） |  |
| At least 6 months | 318（20.4） | 269（27.1） | 49（8.7） |  |
| Admission to the ICU ,n(%) |  |  |  | 0.653 |
| No | 1355（87.1） | 861（86.8） | 494（87.6） |  |
| Yes | 201（12.9） | 131（13.2） | 70（12.4） |  |
| Unexplained infections ,n(%) |  |  |  | 0.555 |
| No | 1389（89.3） | 889（89.6） | 500（88.7） |  |
| Yes | 167（10.7） | 103（10.4） | 64（11.3） |  |
| Cryptococcal meningitis,n(%) |  |  |  | 0.991 |
| No | 1534（98.6） | 978（98.6） | 556（98.6） |  |
| Yes | 22（1.4） | 14 (1.4） | 8（1.4） |  |
| PCP ,n(%) |  |  |  | 0.972 |
| No | 1042（67.0） | 664（66.9） | 378（67.0） |  |
| Yes | 514（33.0） | 328（33.1） | 186（33.0） |  |
| Invasive fungal infections ,n(%) |  |  |  | 0.049 |
| No | 1090（70.1） | 712（71.8） | 378（67.0） |  |
| Yes | 466（29.9） | 280（28.2） | 186（33.0） |  |
| Pulmonary tuberculosis ,n(%) |  |  |  | 0.210 |
| No | 1267（81.4） | 817（82.4） | 450（79.8） |  |
| Yes | 289（18.6） | 175（17.6） | 114（20.2） |  |
| Cytomegalovirus infections ,n(%) |  |  |  | 0.641 |
| No | 1243（79.9） | 796（80.2） | 447（79.3） |  |
| Yes | 313（20.1） | 196（19.8） | 117（20.7） |  |
| Mycobacterium avium complex infections, n(%) |  |  |  | 0.659 |
| No | 1297（83.4） | 830（83.7） | 467（82.8） |  |
| Yes | 259（16.6） | 162（16.3） | 97（17.2 ） |  |
| Systemic multiple OIs,n(%) |  |  |  | 0.701 |
| No | 777（49.9） | 499（50.3） | 278（49.3） |  |
| Yes | 779（50.1） | 493（49.7） | 286（50.7） |  |
| Non-aids-defining events ,n(%) |  |  |  | 0.462 |
| No | 1505（96.7） | 957（96.5） | 548（97.2） |  |
| Yes | 51（3.3） | 35（3.5） | 16（2.8） |  |
| Multiple OIs in respiratory system ,n(%) |  |  |  |  |
| No | 961（61.8） | 619（62.4） | 342（60.6） | 0.492 |
| Yes | 595（38.2） | 373（37.6） | 222（39.4） |  |
| Multiple OIs of the CNS,n(%) |  |  |  |  |
| No | 1479（95.1） | 943（95.1） | 536（95.0） | 0.983 |
| Yes | 77（4.9） | 49（4.9） | 28（5.0） |  |
| Systemic disseminated tuberculosis ,n(%) |  |  |  |  |
| No | 1451（93.3） | 929（93.6） | 522（92.6 ） | 0.407 |
| Yes | 105（6.7） | 63（6.4） | 42（7.4） |  |
| Syphilis,n(%) |  |  |  |  |
| No | 1152（74.0） | 730（73.6） | 422（74.8） | 0.594 |
| Yes | 404（26.0） | 262（26.4） | 142（25.2） |  |
| Length of hospital stay,days (IQR) | 21.14(10.00, 31.00) | - | - |  |

Note：CNS: Central nervous system, HAART: highly active anti-retroviral therapy, ICU: intensive care unit,IQR: interquartile range, LOS: length of stay, MAC: mycobacterium avium complex, OIs: opportunistic infections, PCP: pneumocystis carinii pneumonia, Yes: Discharge diagnosis of this disease, No: Without this disease.
