# Supplementary material for: Supervised machine learning algorithms to predict the duration and risk of long-term hospitalization in HIV-infected individuals: a retrospective study
Source: Front Public Health. 2024 Jan 5;11:1282324. doi: 10.3389/fpubh.2023.1282324 (PMC10796994; doi:10.3389/fpubh.2023.1282324)
Supplement: Supplementary file 3 [file Table_3.docx]

**Table S3:** **Included variables base on the evaluation of variable importance.**

|  | Variable |
| --- | --- |
| 1 | Baseline CD4 cell count |
| 2 | Baseline viral load |
| 3 | Unexplained infections |
| 4 | Admission to the ICU |
| 5 | Cryptococci meningitis |
| 6 | Systemic multiple opportunistic infections(OIs) |
| 7 | Multiple opportunistic infections of the CNS |
| 8 | Non-aids-defining events(NADEs) |
| 9 | Systemic disseminated tuberculosis |

Note: CNS: Central nervous system, ICU: intensive care unit, NADEs: Non-aids-defining events, OIs: opportunistic infections
