# Supplementary material for: Supervised machine learning algorithms to predict the duration and risk of long-term hospitalization in HIV-infected individuals: a retrospective study
Source: Front Public Health. 2024 Jan 5;11:1282324. doi: 10.3389/fpubh.2023.1282324 (PMC10796994; doi:10.3389/fpubh.2023.1282324)
Supplement: Supplementary file 4 [file Table_4.docx]

**Table S4:** **Area under the ROC and PR curves of the classification models**

|  |  | **RF(95% CI)** | **KNN(95% CI)** | **SVM(95% CI)** | **NN(95% CI)** | **XGB(95% CI)** |
| --- | --- | --- | --- | --- | --- | --- |
| **AUROC** | Training set | 0.9315(0.9102,0.9528) | 0.9305(0.9105, 0.9505) | 0.9518(0.9391,0.9645) | 0.9739(0.9613,0.9865) | 0.9674(0.9550,0.9798) |
|  | Testing set | 0.9315(0.9010,0.9619) | 0.9225(0.8889,0.9561) | 0.9419(0.9208,0.9629) | 0.9779(0.9625,0.9934) | 0.9695(0.9510,0.9880) |
| **AUPRC** | Training set | 0.896 | 0.862 | 0.691 | 0.765 | 0.678 |
|  | Testing set | 0.755 | 0.643 | 0.679 | 0.773 | 0.712 |

Note: CI: confidence interval, RF: Random Forest, SVM: Support Vector Machine, KNN: k-Nearest Neighbor, NN: Neural Network, XGB: Extreme Gradient Boosting, ROC: receiver operating characteristic, PR: precision recall curve, AUPRC: area under precision recall curve, AUROC: area under receiver operating characteristic curve
