# Supplementary figures and images for: Supervised machine learning algorithms to predict the duration and risk of long-term hospitalization in HIV-infected individuals: a retrospective study
Source: Front Public Health. 2024 Jan 5;11:1282324. doi: 10.3389/fpubh.2023.1282324 (PMC10796994; doi:10.3389/fpubh.2023.1282324)

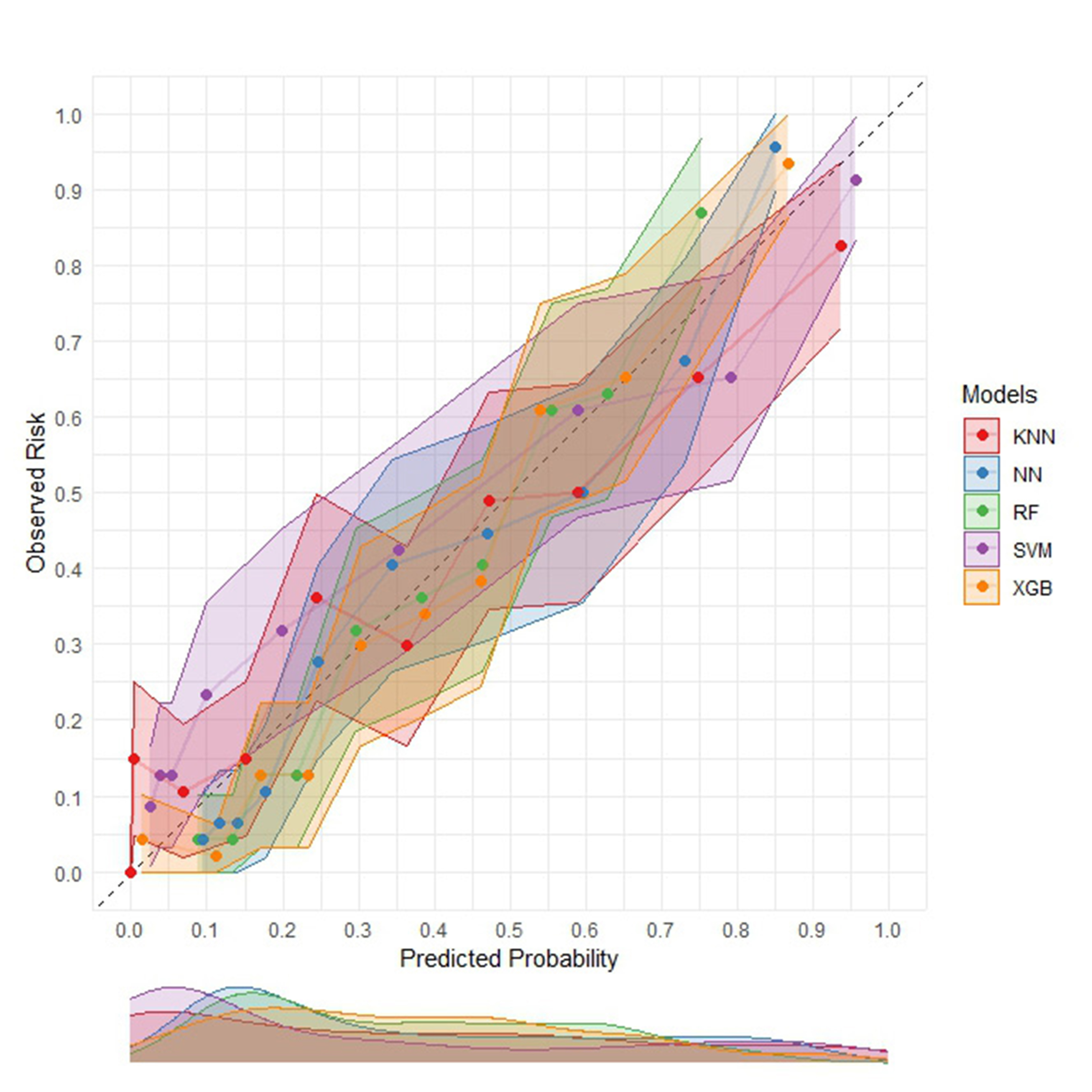

Supplement: Supplementary Figure S1 — Calibration curves for five classification ML models in internal validation (RF, KNN, SVM, NN, and XGB) to predict risk of prolonged hospital stay. [file Image_1.JPEG]

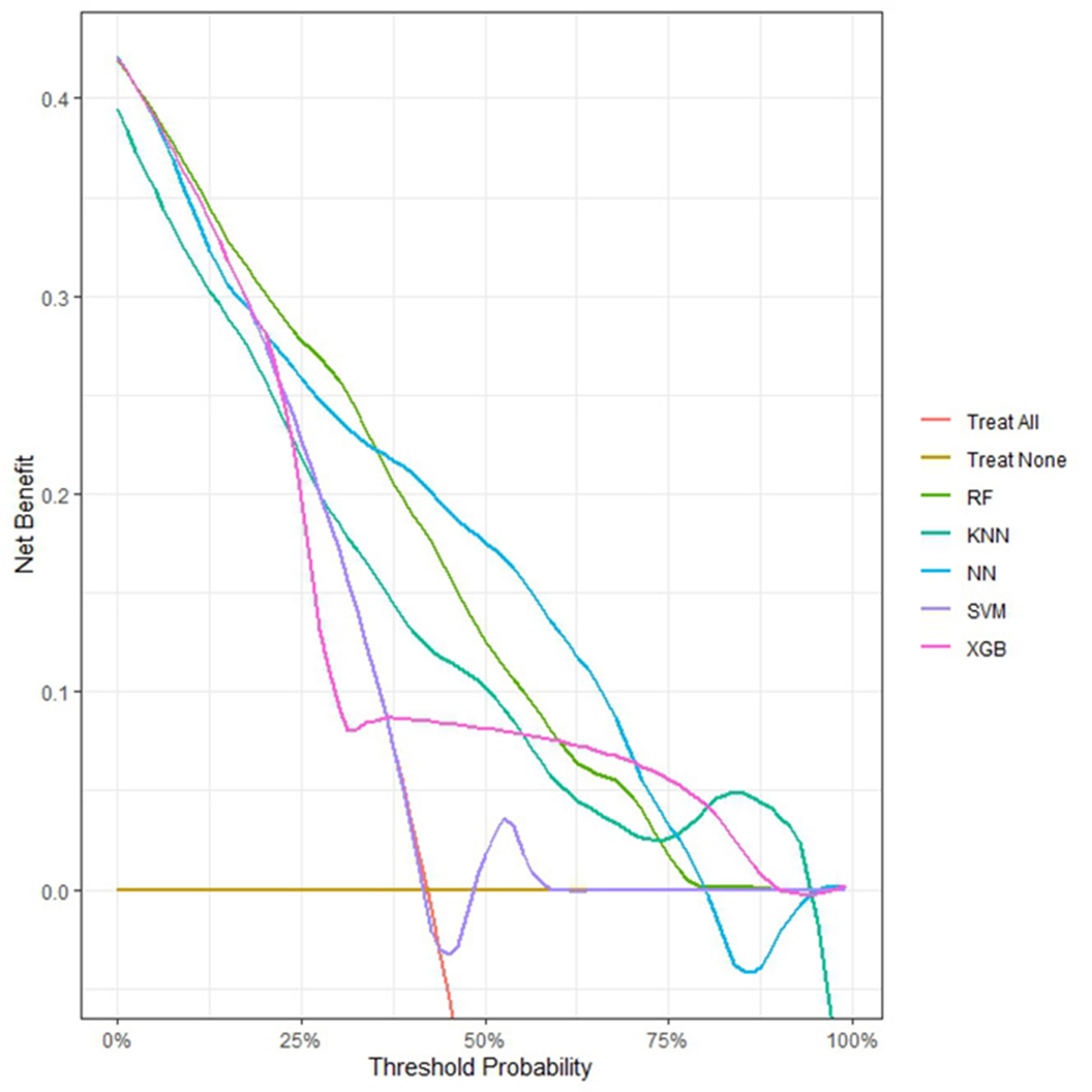

Supplement: Supplementary Figure S2 — Decision curves for five classification ML models in internal validation (RF, KNN, SVM, NN, and XGB) to predict the risk of prolonged hospital stay. [file Image_2.JPEG]
